# High-resolution densitometry and elemental analysis of tropical wood

**DOI:** 10.1007/s00468-014-1126-7

**Published:** 2014-11-27

**Authors:** Peter Hietz, Monika Horsky, Thomas Prohaska, Ingeborg Lang, Michael Grabner

**Affiliations:** 1 Institute of Botany, University of Natural Resources and Life Sciences Vienna, Gregor Mendel Strasse 33, 1180 Vienna, Austria; 2 Division of Analytical Chemistry, Department of Chemistry, University of Natural Resources and Life Sciences Vienna, Konrad-Lorenz-Straße 24, 3430 Tulln, Austria; 3 Core Facility Cell Imaging and Ultrastructure Research, University of Vienna, Althanstraße 14, 1090 Vienna, Austria; 4 Institute of Wood Science and Technology, University of Natural Resources and Life Sciences Vienna, Konrad Lorenz-Straße 24, 3430 Tulln, Austria

**Keywords:** Crystals, Dendrochemistry, Tropical wood, Wood anatomy, Wood chemical composition

## Abstract

****Key message**:**

**Understanding the mobility and distribution of chemical elements in wood is necessary to apply dendrochemistry. Crystals are likely stable and could be used to analyze changes in nutrient supply.**

**Abstract:**

Dendrochemistry uses the variation in wood chemical composition to infer about past environmental conditions and possible effects on tree growth. Elemental or isotopic variation might also help to identify annual growth where tree rings are anatomically not distinct. However, most elements are—to a certain degree—mobile within wood and may be related to anatomical structures. Therefore, understanding what affects elemental distribution is important to make use of and critically assess the potential of dendrochemistry. We studied the variation of wood density and elements at high spatial resolution in wood of six species with anatomically distinct to rather indistinct tree rings from a Thai monsoon forest. Many elements had a higher concentration in parenchyma than in fiber cells, and the co-variation of elements differed strongly between elements and also between species. Strong wood density changes along the ring boundary were found only in *Melia azedarach*. In all species, the X-ray images showed crystals. EDX spectra showed that these consist of calcium or silicon (in *Chukrasia tabularis*) as major elemental components. A high concentration of heavy metals (Fe, Cu and Zn) was found in *Vitex peduncularis*. We conclude that at least for the species studied the radial variation of elemental concentration is unlikely to reveal annual rings that anatomy could not. However, if elements in crystals are more stable than in cell walls or living protoplasts, analyzing the distribution of elements present in crystals may show environmental conditions that, in turn, influence crystal formation and are little known.

## Introduction

Wood represents an important archive, deposited during a tree’s life and reflecting tree physiology and environmental conditions that affect growth. Annual growth in trees with anatomically distinct rings has long been used to measure growth trajectories and as a climate proxy (Speer, 2010). Apart from the total growth, other details in wood anatomy such as vessel size and frequency (Fonti et al. 2010), the proportion of earlywood and latewood or wood density (Büntgen et al. 2010) can provide additional information and more details.

Dendrochemistry analyzes radial variation in wood chemical composition. Variations in elemental and isotopic composition have been used to identify changes in the environment (Augustin et al. 2005; Barrelet et al. 2008). In some cases, the analysis of a highly resolved chemical variation can also help to identify annual growth where annual rings are anatomically not distinct enough (Evans and Schrag 2004; Poussart et al. 2006; Verheyden et al. 2004). This approach has been made possible by instrument developments, in particular laser ablation (LA), which coupled with inductively coupled plasma mass spectrometry (ICP-MS; Hoffmann et al. 1994; Prohaska et al. 1998) or gas chromatography-isotope ratio mass spectrometry (GC-IRMS; Schulze et al. 2004) can achieve a spatial resolution in the µm range.

Any dendrochemical approach depends on the behavior and the mobility of elements or isotopes in the wood. For C and O atoms in cellulose, there is little doubt that these do not move once cell wall formation is finished after a few weeks and LA GC-IRMS is used to analyze inter-annual variation (Schulze et al. 2004). For all other elements, the immobility is questionable. Nitrogen, which is present in the cell wall in the form of proteins, appears to be rather immobile although there is some radial translocation: tracer studies have shown that a pulse of ^15^N is visible as a peak in the ring deposited in the year the tracer was supplied but some spread to the previous and following years was also seen (Elhani et al. 2003; Hart and Classen 2003). Treating wood samples with organic solvents extracts the more labile N, leaving the more stable N in cell walls, which should be less affected by translocation (Sheppard and Thompson 2000). While ^15^N may therefore not be useful to identify year-to-year variation, long-term trends in forest N supply resulting from N deposition or forest regeneration have been shown (Hietz et al. 2010; McLauchlan et al. 2013).

Cations in the cell wall are convertibly bound to negative groups in pectins and their exchangeability and radial mobility depend on the charge, the size, the concentration of other cations including protons, and the presence of a solvent (water). Generally, heavy metals, being large and with a positive charge of two or more, should be relatively stable and radial trends in the concentration of Mn, Cr, Cd, Ni and Cu have been used to show increasing pollution or soil acidification (Augustin et al. 2005; Bilodeau Gauthier et al. 2008; Leonelli et al. 2011). It was suggested that the less extractible, more immobile nutrients can be used to document pollution trends from tree rings (Penninckx et al. 2001) but these can also be misleading when elements accumulate over the years. In this case, a decrease in concentrations towards the most recent years would not indicate a decrease in availability (Watmough and Hutchinson 2002). Here, elemental ratios such as Ca/Sr are better proxies of soil status (Bullen and Bailey 2005) and a careful interpretation of trends seen in tree rings is essential.

In sapwood, which still contains living cells, radial transport can be active. Heartwood formation is also an active process and involves the movement of nutrients (Spicer 2005), reflecting the fact that elemental concentrations in sapwood and heartwood often differ (Meerts 2002). Changes along the sapwood/heartwood boundary can be sharp, as seen in the steep decline of K concentrations from sapwood to heartwood in *Quercus robur* (Smith et al. 2014) or the peak of Ca at the sapwood/heartwood boundary in *Miliusa velutina* (Poussart et al. 2006). As long as wood is live sapwood, many active processes can affect the deposition, exchange and transport (including radially) of elements. Heartwood no longer contains living cells that can actively affect transport, does no longer transport water axially with sap flow; water content is often lower than in sapwood, and it can be impregnated with extractives, so the radial mobility of elements in heartwood should be lower. Still, rubidium injected into sapwood had diffused into heartwood after 10 days (Okada et al. 2012) suggesting that the heartwood concentration of alkaline elements reflects active processes during heartwood formation and passive diffusion afterwards. Diffusion within heartwood should be much slower for elements with higher charges, which will be bound more strongly to the cell wall, compared to alkaline elements.

For Ca, a strong cyclic radial variation has been found in *Miliusa velutina*. This variation appears to be annual and, if widespread, could be used to identify indistinct rings in tropical trees (Poussart et al. 2006). In addition, the annual Ca peak was correlated to rainfall in March in the seasonal climate in Thailand. This is remarkable because water taken up from the soil is transported in sapwood, so the entire sapwood should be exposed to the seasonal variation in Ca availability and not only the newly produced wood. If Ca concentrations in wood reflect the seasonal availability in the soil water or seasonal uptake, this suggests very little mobility, active or via diffusion, over the years. It was also suggested that a seasonal Ca signal in wood might be due to the deposition of Ca-rich crystals or seasonal variation in the amount of cell wall pectins (Poussart et al. 2006), but this was not verified.

Alternatively, some patterns of elemental distribution in wood might not result from external availability but from active radial transport in the symplast or from differences in wood structure. For instance, the concentration of cations can depend on the density of pectins with negatively charged carboxyl groups and may thus reflect wood chemical composition or different types of cells. Since primary cell walls have higher concentrations of pectins than secondary walls (Sarkar et al. 2009), cell types with thick secondary walls such as fibers should have a lower proportion of pectins and cations binding to these than thin-walled parenchyma cells. Mineral inclusions in wood have high concentrations of specific elements, they are not distributed uniformly and can be seen in micro-density measurements (Vansteenkiste et al. 2007) and chemically analyzed by energy-dispersive X-ray fluorescence (Smith et al. 2014). The concentration of nutrients essential for the functioning of living cells will depend on the proportion of living (parenchyma) cells in wood, which often decreases from the cambium onwards and is zero in heartwood. As a consequence, elements such as N, P, K and Mg can show strong decreases from the cambium towards older years (Bullen and Bailey 2005; Lévy et al. 1996).

To apply dendrochemistry in a broader field, we clearly need more information on the behavior of elements in wood. How mobile are different elements, what affects their deposition or transport and what is their cellular location? To address these questions, we measured wood density with high spatial resolution by X-ray densitometry, which showed small elements of high density, which we supposed to be crystals. We, therefore, analyzed the chemical composition of crystals and other anatomic structures with energy-dispersive X-ray microanalysis (EDX) and measured the radial elemental variation by LA inductively coupled plasma mass spectrometry (LA-ICP-MS) to see if the distribution of crystals or other structures can explain the radial distribution of elements in wood. As we aimed to use dendrochemistry for the identification of annual growth, this was tested in six tropical species from a seasonal climate with anatomically distinct to difficult-to-identify annual growth rings.

We expected elemental concentrations to be generally higher in living parenchyma cells than in cell walls of dead cells. Thus, concentrations should also be lower in heartwood without living cells than in sapwood. Since outside living protoplasts the distribution should be mainly driven by diffusion and ion exchange, we expected elements of similar charge to show similar fine-scale distribution within wood. We finally expected that if seasonal variations in elemental concentrations were found at all, these would be due to the distribution of elements in crystals, which do not easily dissolve, and not in cell walls, where ions can always be exchanged.

## Materials and methods

Samples were collected at the 50 ha permanent study plot in the Huai Kha Khaeng Wildlife Sanctuary in Uthai Thani province, west-central Thailand (15°09′N, 99°10′E; Bunyavejchewin 1999). The area has a monsoonal climate with approx. 1,500 mm annual rainfall and <100 mm per month in the dry season between November and April. Mean monthly temperature is highest in July (27 °C) and lowest in January (19 °C). The forest is classified as seasonal dry evergreen forest, though a few species shed their leaves during the dry season. Since the main purpose of the project was tree-ring analysis, we selected species known to produce anatomically distinct tree rings: *Toona ciliata* M. Roem., *Melia* *azedarach* L. and *Chukrasia tabularis* A. Juss (Meliaceae), *Afzelia xylocarpa* Craib (Fabaceae), *Neolitsea* *obtusifolia* Merrill (Lauraceae), *Vitex peduncularis* Wall. ex Schauer (Verbenaceae). Those of three Meliaceae were the most distinct and cross dated (Nock et al. 2011), those of the other three species, while still anatomically distinct, were more difficult to distinguish from possible false rings and cross dating was difficult. Tree increment cores were collected with a 5 mm borer.

### Density measurements

For X-ray densitometry, air dry cores were sawn with a double-bladed saw to a thickness of 1.4 mm. Samples and cellulose acetate density standards were exposed to X-rays for 25 min. Developed films were scanned using a specially designed scanner with a motor and a line camera on a light microscope (Stemi 2000, C. Zeiss, Jena, Germany; Grabner et al. 2005). The final resolution of the scanned images was about 10 μm, which is similar to the grainy structure the X-ray film yields. Radial variation of wood density of the cores had been analyzed from 9 to 26 trees per species (Nock et al. 2011). To identify the role of density as a result of extractives, one sample per species was placed in 1 N HCl for 24 h. We used HCl instead of acetone because we were primarily interested in the identity of the high-density elements, which, if Ca crystals, should dissolve in HCl. After extraction, densities were measured again as described above.

X-ray density images were analyzed with SigmaScan Pro 5.0 (Systat Software Inc., Chicago, USA). Radial density gradients were measured by drawing a line along the rays. The “Trace”-option in SigmaScan measures the brightness of a line between 1 and 50 pixels wide, recording *x* and *y* position of the center of the line. We used a line thickness between 5 and 50 pixels, where a thin line captures more detail and gradients may turn out sharper but produces a noisier signal. Most images showed small elements of very high density. After improving the contrast with a Sobel filter, these small high-density elements were defined by a minimum size of five pixels and measured as above.

### Chemical microanalysis

We used energy-dispersive X-ray (EDX) microanalysis to investigate the distribution of elements in distinct anatomical features such as the walls of different cell types and crystals. EDX is a semi-quantitative method and the elemental content is detected in relation to all other elements analyzed in the sample; in our case C, N, O, Na, Mg, Al, Si, P, S, Cl, Cd, K, Ca, Fe, Ni, Cu and Zn.

We selected one or two samples per species, made a fresh cut of the cross section with a razor blade and mounted them on 0.5′′ aluminum specimen stubs equipped with sticky carbon foil (Agar scientific, UK) and carbon coated (Sputter coater EPA 101) to prevent surface charge.

EDX was performed on a Philipps XL 20 scanning electron microscope (SEM). For data collection, background subtraction and element specific spectra analyses, we used EDAX-Genesis software Version 5.11 (Ametek Material Analysis, USA) which fully deconvolves the spectra and allows the corrections for interference between elements. Microanalysis was performed at an acceleration voltage of 30 kV with a working distance of 12 mm (sample to final lens); tilt was 15°, take-off angle 27.21° and the dead time was approximately 30 percent. Analyses were performed at a magnification of 1,000–5,000× on crystals or spot area of selected cells. Multiple measurements per sample were taken.

### Laser ablation ICP-MS

We used laser ablation ICP-MS to test if the radial variation of elemental concentration reflects anatomically distinct growth rings. The surface of increment cores was polished with sanding paper up to grit 600 and the wood powder was removed with pressurized air. One wood sample per species, representing the outermost centimeters inside the cambium, was fixed onto glass slides using plasticine and inserted into the sample cell of the laser ablation system NWR193 (Electro Scientific Industries, Inc., Portland, USA), which was coupled to an inductively coupled plasma mass spectrometer (ICP-MS; Nexion 300D, Perkin Elmer, Waltham, USA). A laser beam is focused onto the sample surface and ablates small quantities of the sample while scanning over its surface along the length of a core. The particles are then transported to the ICP-MS by a carrier gas flow, where atomisation, ionization and separation according to the mass-to-charge ratio occur. Operating parameters are summarized in Table 1. Prior to each ablation, a gas blank was recorded for at least 60 s. Lines on the drill core samples were between 11 and 26 mm long.

**Table 1:** LA-ICP-MS operating parameters

Laser ablation system NWR193	
Wavelength (nm)	193
Scan type	Line scan
Spot size (μm)	150
Scan speed (µm s^−1^)	30
Fluence (J cm^−2^)	12.4
Frequency (Hz)	20
Carrier gas flow (He) (L min^−1^)	0.9
ICP-MS (Nexion 300D)	
Mix gas flow (Ar) (L min^−1^)	1
Auxiliary gas flow (L min^−1^)	1.2
Plasma gas flow (L min^−1^)	18
ICP RF power (W)	1,400
Detector mode	Dual
Monitored isotopes*	^13^C, ^**23**^ **Na**, ^24^Mg, ^**26**^ **Mg**, ^**31**^ **P**, ^**39**^ **K**, ^42^Ca, ^**43**^ **Ca**, ^44^Ca, ^54^Fe, ^56^Fe, ^**57**^ **Fe**, ^**60**^ **Ni**, ^62^Ni, ^**63**^ **Cu**, ^65^Cu, ^**66**^ **Zn**, ^68^Zn, ^**85**^ **Rb**, ^**88**^ **Sr**, ^**208**^ **Pb**

* Data are presented only for isotopes shown in boldface

Corresponding gas blank averages were subtracted from all recorded signals. Then, a moving average over five points each was calculated and the obtained signals were normalized to the intensity of ^13^C to correct for variations in ablated mass. For each element, one isotope was chosen for further data evaluation based on high signal intensity, low limit of detection (calculated as three times the standard deviation of the gas blank signal) and absence of interferences. Because we are interested in comparing spatial variation between elements rather than in absolute concentrations, all LA-ICP data were standardized by dividing by the mean, so that the mean of any element in a sample is 1.

We calculated principal component analyses (PCA) for the LA-ICP-MS results to show to what extent the distribution of various elements and isotopes is similar to each other or vary independently. PCA was calculated with R 2.15.2 (R foundation for Statistical Computing, Vienna, Austria).

## Results

**Fig. 1:**
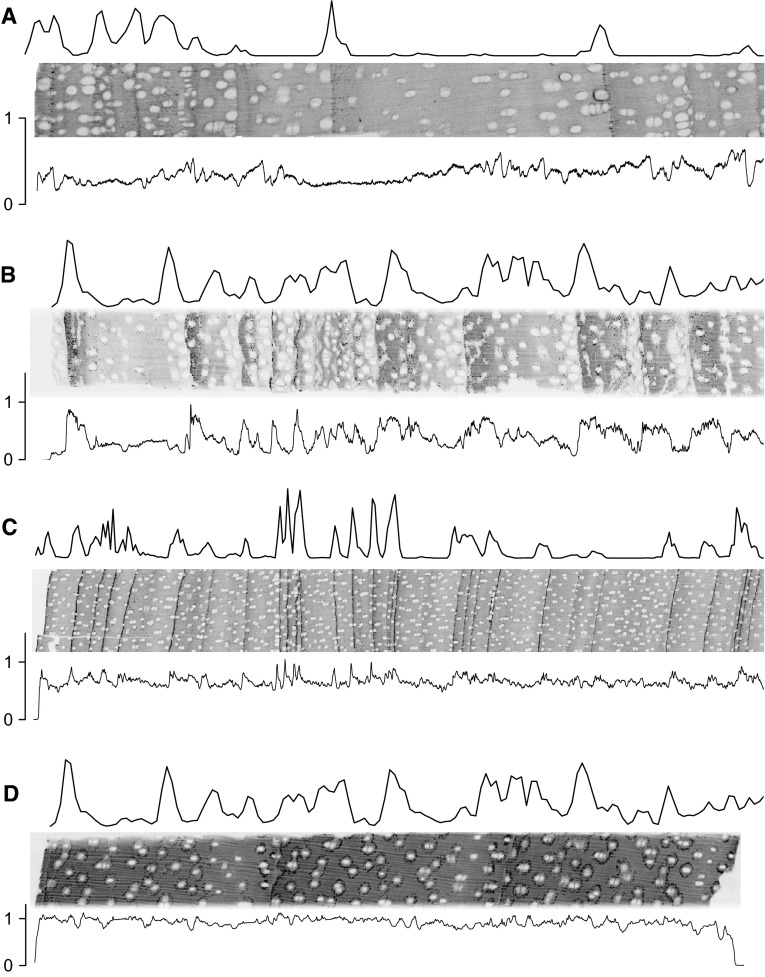
X-ray images of *Toona ciliata* (**a**) *Melia azedarach* (**b**), *Chukrasia tabularis* (**c**), and *Afzelia* *xylocarpa* (**d**). The X-ray image was inverted so that denser parts appear *darker*. The *line above* the X-ray images shows the relative abundance of small high-density elements (visible as black dots on the X-ray image) smoothed with kernel density estimation and a bandwidth of 10–50. For *Chukrasia*, densities were weighted by element size as dark elements were often confluent. The *line below* the image is the wood density (g cm^−3^) over a width of 200 pixels (the entire image is c. 550 pixels high). The cambium is to the left of the images

**Fig. 2:**
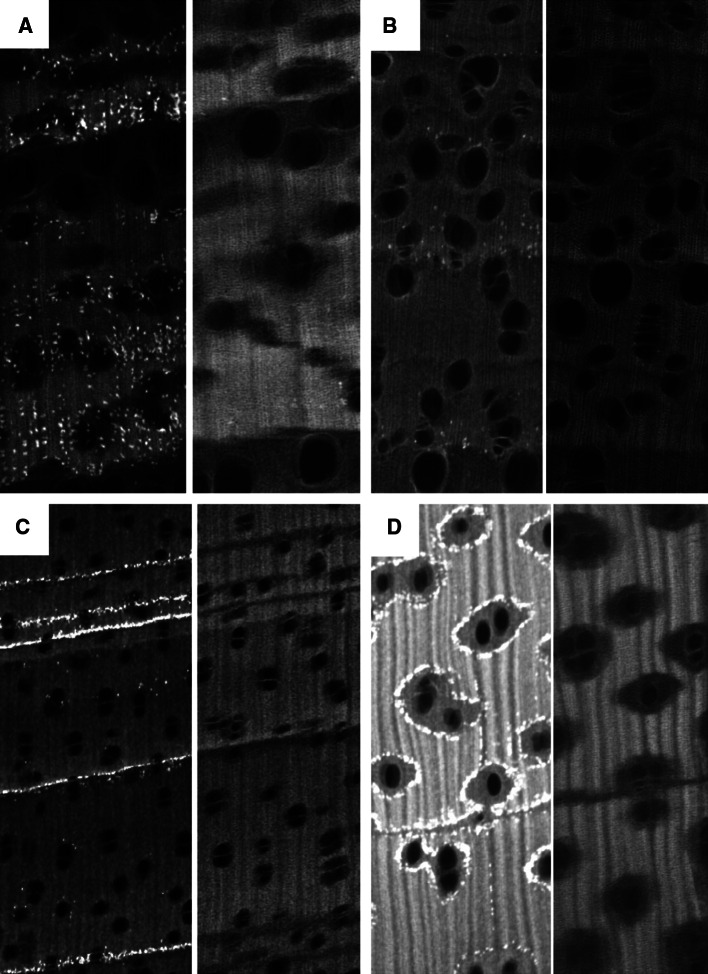
Comparison of X-ray images of samples of untreated wood (*left half of image*) and extracted with HCl (*right half*). **a** *Toona ciliata*, **b** *Melia azedarach*, **c** *Chukrasia tabularis* **d** *Afzelia xylocarpa*

X-ray images clearly showed the vessels, which to some extent affect micro-densities measured along a line c. 200 μm wide (Fig. 1). Ring boundaries were mostly identified by thin parenchyma bands. In *Toona*, ring boundaries are also characterized by larger vessels in earlywood; in *Melia*—and to a lesser extent in *Toona* and *Afzelia*—also by abrupt changes in wood density. Furthermore, the X-ray images showed small elements of very high densities (Fig. 2). In *Chukrasia*, these are numerous and strongly concentrated on ring boundaries; in *Vitex* (not shown), they are similar to *Chukrasia* but less numerous; in *Melia*, mostly in ring boundaries but also seen within an annual ring; in *Toona*, less numerous, mostly on ring boundaries but also in between. In *Afzelia*, crystals are very common, sometimes along ring boundaries but particularly forming rings around the paratracheal parenchyma. *Neolitsea* shows sometimes abrupt, sometimes more gradual variations in wood density but no small elements of high density were observed (not shown). Treatment with 1 N HCl in all cases removed the high-density elements (Fig. 2).

**Fig. 3:**
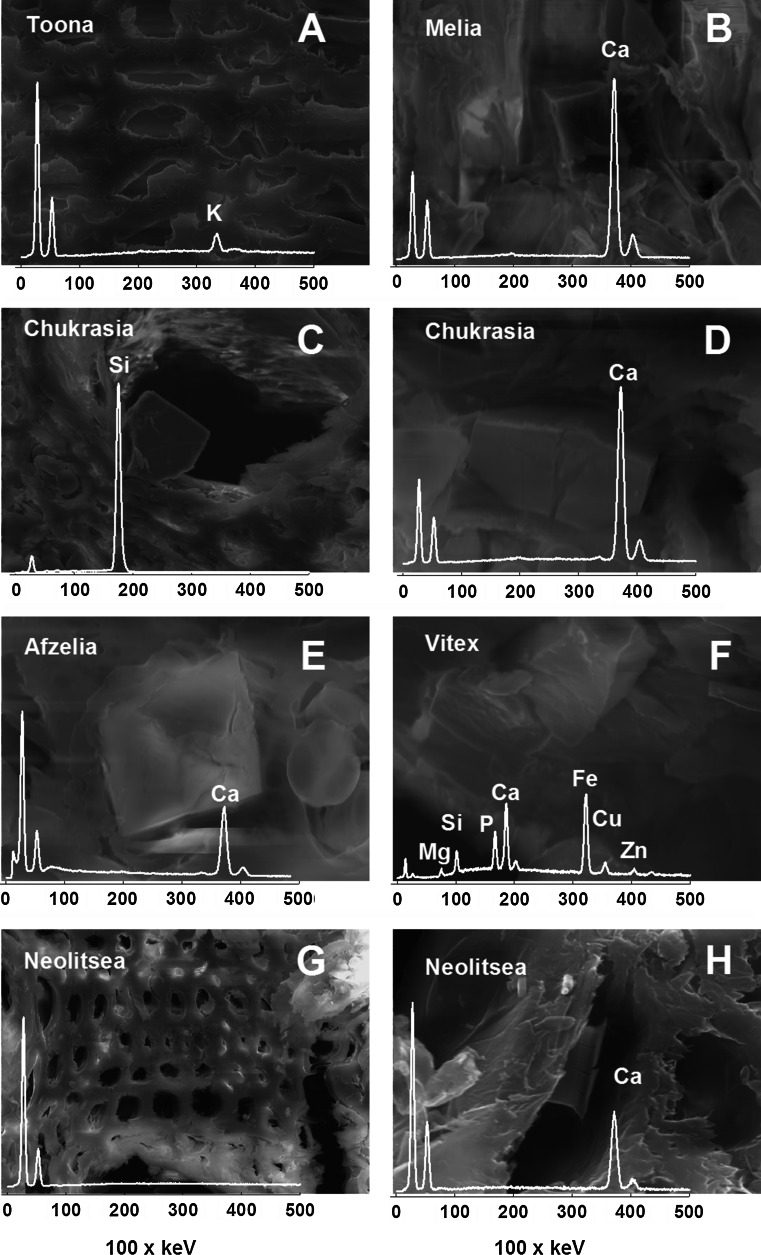
SEM images and EDX spectra of wood parenchyma cells with different crystals (*Melia* *Chukrasia, Afzelia*, *Vitex* and *Neolitsea*) or fiber cells (*Toona, Neolitsea*)

In all species, crystals were seen on SEM micrographs (Fig. 3). EDX spectra show that in most species these crystals contained predominantly Ca. *Chukrasia* exhibited also crystals with Si. Crystals in *Vitex* had a high concentration of Fe, in addition to Ca, K, P, Cu and Zn. Elemental concentrations in cell walls measured in fibers and parenchyma cells were mostly low but sometimes showed peaks in K or Ca (e.g., *Toona* in Fig. 3).

**Fig. 4:**
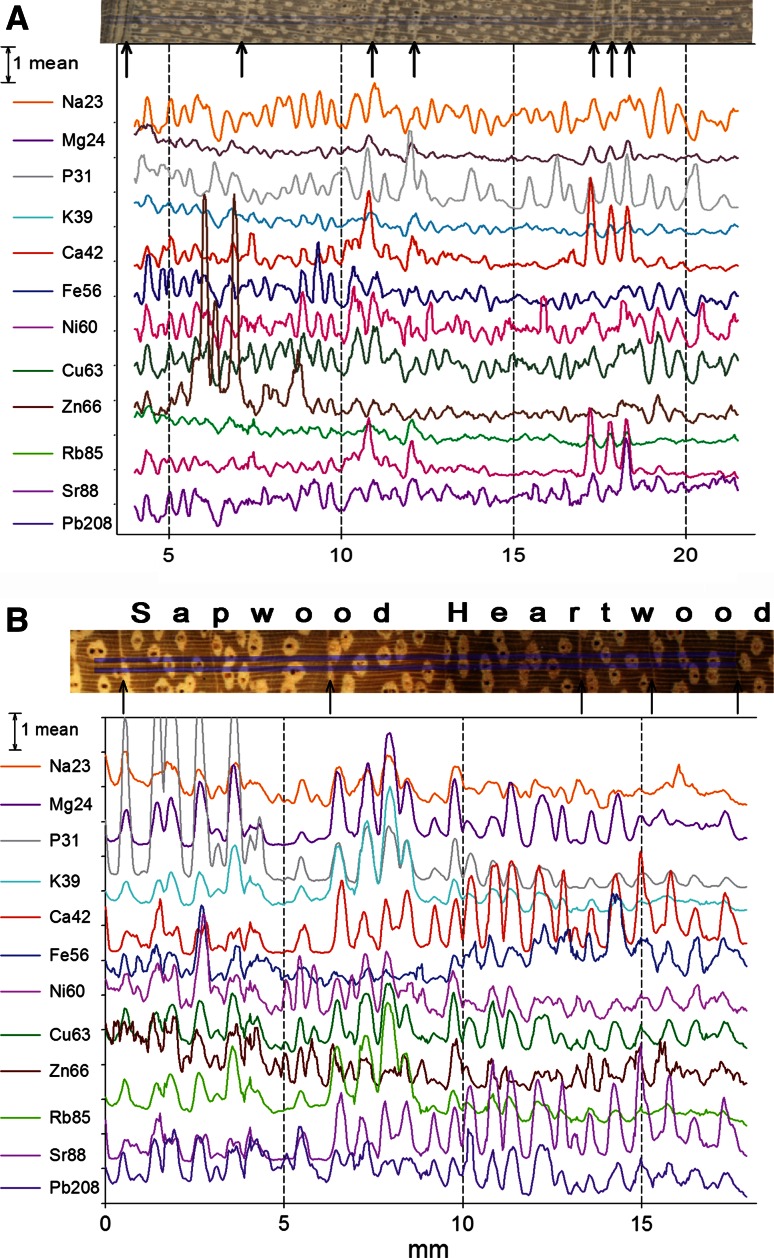
Variation in element concentrations along a radial profile in wood of *Chukrasia tabularis* (**a**) and *Afzelia xylocarpa* (**b**). Concentrations are relative and scaled to the mean of each element. The laser ablation line is between the two parallel *blue lines* on the image. The *arrows *mark parenchyma bands of putative annual ring boundaries. In *Afzelia*, heartwood is distinguished from sapwood by its *darker color*

Figure 4 shows the radial variation in elemental concentrations. *Afzelia* has large paratracheal parenchyma groups around the large vessels so that the 150 μm resolution of the laser ablation distinguishes between these living cells and wood dominated by dead fibers where parenchyma is only present in narrow rays. Elemental concentrations were generally higher in parenchyma tissue than in fibers. The laser ablation line in *Afzelia* also captured the transition between lighter sapwood and darker heartwood. For P, K, and Rb and to some extent for Mg and Ni, concentrations in heartwood were lower than in sapwood, particularly in the (dead) parenchyma. In *Chukrasia*, paratracheal parenchyma is smaller than in *Afzelia* and the variation in elemental concentrations can therefore not clearly be related to the tissue type. In *Chukrasia*, tangential parenchyma bands that mark annual growth rings are most distinct and have the highest concentrations of Ca and Sr. The *Chukrasia* sample consisted entirely of sapwood, but the concentrations of Mg, P, K, Fe and Rb decreased from the cambium to the inner wood. The adjacent bark was also measured but concentrations are not shown as these were mostly much higher than in wood and would be off the scale.

**Fig. 5:**
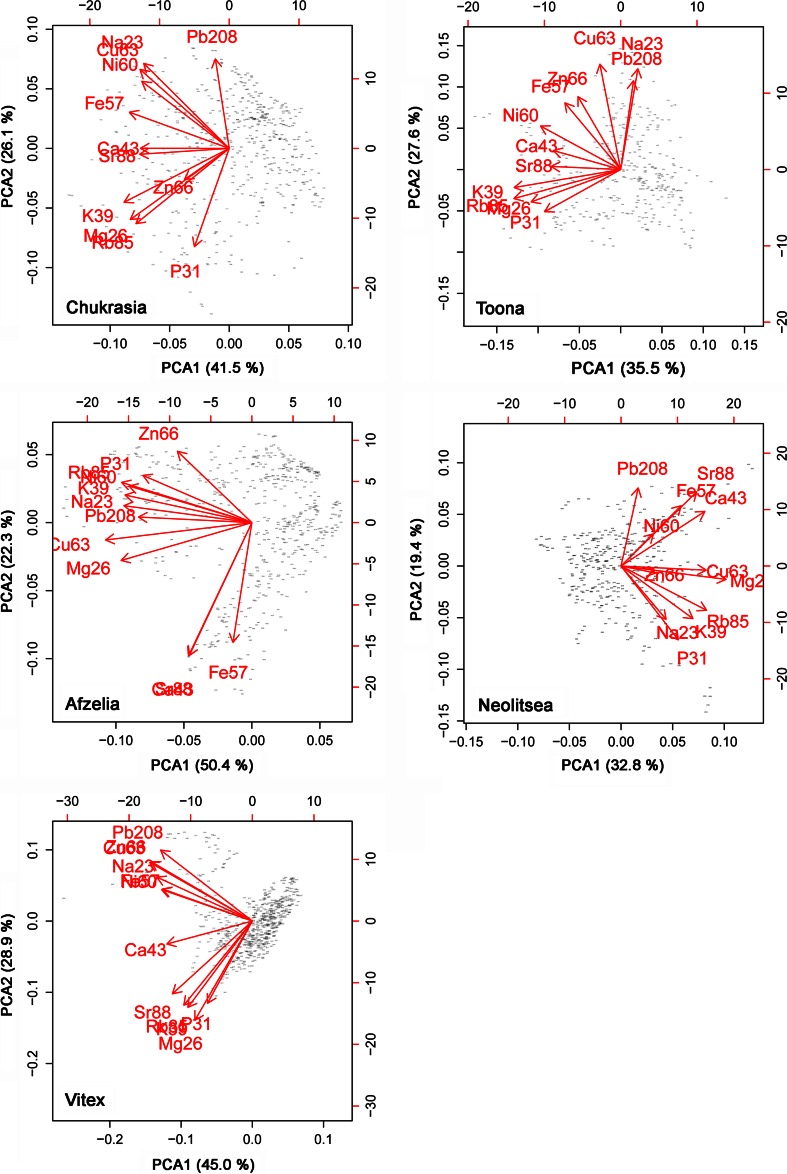
Principal component analysis of elemental distribution in wood of five species from Thailand. PCA1 and PCA2 represent the first and second component, the % values are the proportions of the variances explained by these components, and the *small symbols* represent the individual measurement points along one sample (see Fig. 4)

Generally, all elements scale in one direction on the first PCA axis (Fig. 5), indicating that in most cases the concentration of any two elements is correlated (at least to some extent). Furthermore, the PCA suggests that tissues containing a higher concentration of one element tend to have higher concentrations of other elements as well. The distributions of K and Rb were similar, as were those of Ca and Sr. Heavy metals (Fe, Ni, Cu, Zn, Pb), Na, Ca and Sr tend to group and be opposed to the other elements. There are some exceptions to this. For instance, Zn scales with Mg and other non-heavy metals in *Chukrasia*; in *Afzelia*, Ca, Sr and Fe group separately and heavy metals other than Fe link with the other elements; and in *Neolitsea*, Ca and Sr scale more with heavy metals than with other nutrients.

## Discussion

Wood density can be used to identify annual growth rings in temperate conifers (Polge 1970). In some tropical species, growth rings are also characterized by density variation though terminal parenchyma bands are more common. The X-ray densitometry shows clear density variations in *Melia azedarach* (Fig. 1b), but this does not reveal tree rings better than anatomical inspection would.

Apart from P, which is covalently bound in organic molecules, the elements measured are cations differing in size and charge, some are essential nutrients, and others are not. In most cases, the elements co-vary, which is seen in the often synchronous variation along the radial profiles (Fig. 4) and also supported by the fact that all elements scale in the same direction along the first PCA axis (Fig. 5). The PCA shows that chemically similar elements often scale similarly (Ca and Sr, K and Rb, to a lesser extent the heavy metals Fe, Cu, Zn, Ni, and Pb), irrespective of being essential nutrients or not (Prohaska et al. 1998). Also, PCAs between species differ, particularly in *Vitex* and *Afzelia*. *Vitex* was unusual in containing high concentrations of heavy metals in crystals. *Vitex* sp. and other Verbenaceae have been shown to store high concentrations of Ni in leaves and stems (Fernando et al. 2013), pointing to occasional hyperaccumulation in this family. At least part of these could be deposited in crystals.

The mobility of elements in wood is related to the size and charge of the ion, which may explain why some elements can provide reliable information about changes in supply whereas others do not (Monticelli et al. 2009). However, also for the same heavy metal, results from different studies appear to contradict [see discussion in Monticelli et al. (2009)] and elements behave differently in different species. Knowing in which cases metals are deposited in either cell walls or mineral inclusions might help resolve these contradictory data.

The concentration of elements in wood is strongly related to cell types and anatomical structures. Since secondary cell walls contain few ion exchange sites, tissue with thick cell walls, such as fibers, has low concentrations of most elements (Fig. 3a–g). Parenchyma cells have typically thinner cell walls containing relatively more pectins with ion exchange sites binding cations. In addition, the protoplast in living parenchyma cells contains all essential nutrients. While nutrients are moved through the symplast by active transport, transport in the apoplast outside living parenchyma cells is through diffusion and mass flow of water and is not selective. When the protoplast of parenchyma cells dies during heartwood formation, elements in organic compounds (P, N) may be released by hydrolysis. K is not covalently bound in organic molecules but to ion exchange sites in the cell wall and is also dissolved in the protoplast. For P, K and Rb, the very strong decline in concentration between sapwood and heartwood can only be explained by active withdrawing from senescing wood (*Afzelia* in Fig. 4b), while the concentration of most other elements did not change much between sapwood and heartwood. Interestingly, in *Quercus* also the concentration of the less mobile Ca, Mg and Mn substantially declines in heartwood (Penninckx et al. 2001), indicating a species-dependent difference in the ability to mobilize these nutrients.

For the other elements, a radial trend in elemental concentrations is less clear but their concentrations are generally higher in parenchyma tissue. In *Chukrasia*, the concentrations of Ca and the chemically similar Sr reflect annual growth rings (Fig. 4a). This is because the observed Ca crystals (Fig. 2c) are strongly incorporated in the parenchyma marking the ring boundaries (Fig. 1c).

However, in none of the six species studied does the variation of any element appear useful to identify annual growth rings that could not be seen by anatomical features. Thus, the result from *Miliusa velutina*, also from Thailand (Poussart et al. 2006), could not be replicated in other species. It remains unclear but interesting why Ca concentrations vary seasonally and with climate in that species. Although the Ca availability in soil water and/or Ca uptake and transport in xylem may be related to precipitation, water is transported through sapwood that is active for several years all of which should be affected by the variation in Ca concentrations in xylem sap. The concentration in wood might reflect Ca availability if Ca is bound to ion exchange sites soon after cell wall formation is completed and not exchanged later. However, this is unlikely as fertilization studies have shown that other cations such as Mg and Mn (Houle et al. 2002) are transported through sapwood that was produced long before fertilization. Another study found that the concentration of heavy metals (Cd, Pb and Zn) changes during the year, even in heartwood that was produced many decades from the cambium (Hagemeyer and Schäfer 1995). This can only happen if elements are re-distributed via diffusion, which violates the basic assumption of dendrochemical analysis that requires a stable mineral distribution (Nabais et al. 1999).

As many crystals are rather insoluble, the mobility of elements bound in crystals is likely to be much lower than in cell walls and they should therefore be stable, also in heartwood. Crystal formation appears to be completed soon after xylogenesis as we see no increase in crystal frequency from the cambium inwards (Fig. 1). In species where the size or density of crystal varies seasonally, the variation of elements forming these crystals, in many cases Ca, could be used as dendrochemical proxies for the environment and to identify annual growth. This may explain radial variation of Ca in *Miliusa*, but unfortunately does not appear to be common.

## Conclusions

A basic assumption of dendrochemistry is the relation of wood chemical composition to the time the wood was produced, which requires the radial transport of elements to be minimal or that mobile fraction can be removed. Past studies have shown that dendrochemistry can be a useful document for changes in nutrient supply, principally through deposition or soil acidification, but in other cases the radial mobility of elements would exclude such application. Our study shows that elemental concentration is strongly related to anatomical features. Knowing how and in which form trees deposit elements in wood will help to understand the limits and potential of using the information stored in wood.
